# Body Image, Medication Use, and Mental Health among Women with Fibromyalgia in Flanders, Belgium

**DOI:** 10.3390/ijerph19031418

**Published:** 2022-01-27

**Authors:** Roel Van Overmeire, Lara Vesentini, Stephanie Vanclooster, Emilie Muysewinkel, Johan Bilsen

**Affiliations:** Mental Health and Wellbeing Research Group, Department of Public Health, Vrije Universiteit Brussel, 1090 Brussel, Belgium; lara.vesentini@vub.be (L.V.); stephanie.vanclooster@vub.be (S.V.); emilie.muysewinkel@vub.be (E.M.); Johan.bilsen@vub.be (J.B.)

**Keywords:** body image, mental health, women’s health

## Abstract

Fibromyalgia (FM) is a chronic illness that does not have clear physical consequences, yet research shows that FM patients often have a low body image. An online cross-sectional study was conducted in Flanders, Belgium, among FM women who are connected to the Flemish League for fibromyalgia patients. An adjusted Body Image Scale (BIS) was used to assess body image, the General Health Questionnaire-12 (GHQ-12) was used for mental health, and the Visual Analogue Scale Fibromyalgia Impact Questionnaire (VASFIQ) was used for FM symptoms. Medication use was assessed by using a 4-point Likert scale. Time since diagnosis and age was assessed. A total of 103 women with FM responded. Linear regression showed that BIS was best predicted in a model by using VASFIQ, GHQ-12, time since diagnosis, and sleep medication, wherein only the GHQ-12 was significant as a variable (B = 0.292; *p* = 0.009). This model explained 19.3% of the variance. The role of sleep medication use disappeared when controlling for mental health. Mental health was more clearly associated with body image than medication use, or even fibromyalgia symptoms. Thus, having negative mental health is associated with a negative body image. In order to improve the body image of FM patients, symptom control alone is not enough; improving mental health is equally important.

## 1. Introduction

Fibromyalgia (FM) is a chronic rheumatologic disease that mostly affects women and has a prevalence of around 2–3% in the general population [1,2]. It causes musculoskeletal pain, chronic pain, physical exhaustion, and can be associated with mental health problems [1].

FM does not have clearly visible consequences, apart from bloating [1]. Some medications might also contribute to certain physical consequences (e.g., weight gain), but these are not clearly visible nor are they exclusive to FM [2]. Due to the lack of clearly physical symptoms of FM, FM has been called an “invisible” illness [3]. However, despite there being overall no clear somatic indicators of FM, qualitative research has shown that some women with FM tend to feel that other people can see that they have FM, which causes them to feel ashamed to go outside [3]. This indicates that FM is associated with a negative body image. Body image refers to individuals’ subjective perceptions, feelings, and thoughts about their physical body, and it is part of self-image [4,5].

There are indeed some studies that have shown that FM might be associated with a negative body image [2,4,5,6], yet it remains unclear why women with FM may have a negative body image. While weight does play a role in this perception [7,8,9], one study showed that a negative body image could not be associated with obesity [10]. Therefore, other associations should be explored. A possible association may exist between women’s mental health status and their negative body image, as this association has been shown to possibly play a large role in explaining body image among FM women [4,10,11,12]. However, the association between mental health and body image remains an understudied aspect among FM women [4].

Apart from the association between mental health and body image, there is also the important aspect of medication use. FM women often are treated with certain types of medications: antidepressants for their depressive symptoms, sleep medication to help with sleep disorders associated with FM, or pain medication for chronic pain [13]. These forms of medications can be important confounders in investigating the relationship between body image and mental health. It also has never been investigated what direct role all these types of medications may play in affecting the body image of FM women.

Thus, in this study we have as a hypothesis that there is an association between mental health and medication use on the one hand and body image on the other hand, where negative mental health leads to a negative body image, and medication use leads to a negative body image.

The health of people with chronic illnesses not only involves the absence of pain or other disease-related factors, but it also involves social, emotional, and psychological challenges [14]. While there has been attention for the mental health or sexual health of women with FM [12,15], body image, an important aspect of wellbeing, is mostly unstudied. To achieve more positive health outcomes, all aspects are important to study. Studies on body image are, therefore, important to gain a more profound understanding of the consequences of FM. The knowledge from such studies can also help healthcare workers and therapists treat FM patients more adequately. Thus, in this study, we aim to investigate the body image of women with FM in Flanders, Belgium.

## 2. Materials and Methods

### 2.1. Study Population and Data Collection

An online survey was made available by the Flemish league for fibromyalgia patients (in Dutch: “Vlaamse Liga voor Fibromyalgie Patiënten”) to its members by accessing a link on social media and on their website. Participants could access the survey by using that link. Only adult female patients with a formal diagnosis of fibromyalgia were included, meaning that respondents had to indicate in the questionnaire if they had received such a diagnosis from healthcare personnel, and how long ago this diagnosis was given. If respondents stated that they had not received a formal diagnosis of fibromyalgia, they were left out of analyses. Data were collected between 1 March 2021 and 1 April 2021.

### 2.2. Measures

Body image was measured using an adjusted form of the Body Image Scale (BIS). The original BIS contains 10 items, but the last question “Have you been dissatisfied with the appearance of your scar?” was left out due to its unsuitability for the purpose of this study (see Patel et al. [16] for a similar study design). Consequently, the scale consisted of 9 questions concerning body image (e.g., “Have you felt less physically attractive?”). Respondents could answer with “not at all” (0) to “very much” (3). The total score of this adjusted scale is the sum of all responses, resulting in a range from 0 to 27, with higher scores reflecting lower body images (α = 0.922).

Mental health was measured using the General Health Questionaire-12 (GHQ-12) (α = 0.874). GHQ-12 consists of 12 items that survey an individual’s mental health of the past few weeks (e.g., “In the past few weeks, have you lost much sleep over worry?”). It provides four answer choices and yields scores ranging from 0 to 36, where higher scores are more indicative of mental health problems. We also included a GHQ-12 score between 0 and 12, as this allowed us to compare our averages to a national average [17]. For analyses, a GHQ-12 score between 0 and 36 was used.

Frequency of use of sleep medication, antidepressants, and pain medication was assessed using a 4-point Likert scale. Respondents were asked how often they had used a type of medication in the past two weeks, with answer options from “not at all” to “almost every day”.

Fibromyalgia symptoms were measured using the Visual Analog Scale Fibromyalgia Impact Questionnaire (VASFIQ) (α = 0.762). This is a 7-item scale concerning common FM symptoms (e.g., pain). It has a global score of FM symptoms ranging from 0 to 70, where a higher score indicates more fibromyalgia symptoms [18].

Finally, participants were asked about age and time since the diagnosis of FM. Age was asked using categories, each with a time span of 10 years. The range of time since diagnosis ranged from “1 month or less ago” to “more than 5 years ago”.

### 2.3. Analysis

All analyses were conducted with SPSS 26.0. Firstly, assumptions for linear models were tested using PP plots, multicollinearity tests, and Kolmogorov–Smirnov tests. When necessary, non-parametric methods were used. Secondly, Pearson correlations were conducted between BIS, GHQ-12, and VASFIQ and Spearman correlations were conducted between the previously mentioned variables and ordinal variables such as the three-medication use variables, age, and time since diagnosis. Thirdly, multivariable linear regressions were conducted with body image as outcome. The R2 scores are always adjusted R2 scores. Finally, Mann–Whitney tests were performed for VASFIQ, BIS, and GHQ-12 between participants who used no sleep medication or antidepressants and those that used both, as research shows that there may be a difference between these two groups for mental health [19]. In order to avoid multiple testing problems, we used a significance level of *p* < 0.01 instead of the usual *p* < 0.05.

### 2.4. Ethics

This study was approved by the ethical commission of the UZ Brussels/VUB (B.U.N. 1432021000409). This approval entailed respondents participating anonymously, and they were informed of all their rights in the introduction screen of the survey.

## 3. Results

In total, 103 respondents completed the survey. Most respondents were between 38 and 57 years old (68%), and 49.5% had been diagnosed with FM more than 5 years ago.

Pain medication was used at least once in the past two weeks by 83.5% of the sample, sleeping medication by 47.6%, and antidepressants by 46.6%. The average score for BIS was 14.66 (±7.02) and 49.17 (±10.48) for VASFIQ; 21.24 (±6.08) for GHQ-12 when calculated from 0 to 36; and 7.08 (±2.27) for the GHQ-12-score between 0 and 12 (see Table 1).

BIS was significantly associated with GHQ-12 (r = 0.349; *p* < *0*.001), VASFIQ (r = 0.296; *p* = *0*.002), and the use of sleep medication (rho = 0.272; *p* = *0*.006). Sleep medication use also correlated with antidepressant use (rho = 0.303; *p* = *0*.002) but not with the use of pain medication (rho = 0.213; *p* = *0*.031) (see Table 2).

Linear regression showed that while VASFIQ predicts the BIS significantly in most models, it is not significant when adding GHQ-12. The model with VASFIQ, time since diagnosis, sleeping medication, and GHQ-12 explained the most variance (R2 = 19.3%) (model 6). Only GHQ-12 was significant in this model (B = 0.292; *p* = 0.009). Considering the possible overlap between certain VASFIQ questions on mental health and GHQ-12, we also included a model without VASFIQ included (model 7). In this case as well, GHQ-12 was the only variable that significantly predicted BIS scores (B = 0.376; *p* < *0*.001). This model explained 18.3% of the variance (see Table 3).

Finally, we performed Mann–Whitney tests to compare the group of participants that used both antidepressants and sleep medication (*n* = 30) with the group of participants that used no medication (*n* = 35). There were no significant differences found, although the difference for body image would have been significant had we used a 0.05 *p*-value cutoff. It should also be noted that the scores for the group that used no medication were consistently lower (see Table 4).

## 4. Discussion

We investigated body image among FM women in Flanders, Belgium.

This study showed that there is a significant relationship between having a negative body image and negative mental health. Moreover, fibromyalgia symptoms proved to be related to body image but not when investigated parallel with mental health. Pain medication and antidepressants were not associated with body image. Sleep medication was also significantly associated with a negative body image. However, when controlling for mental health symptoms and FM symptoms, this association disappeared. Medication usage does not seem to be an important predictor compared to mental health, which was further emphasized by the lack of significant differences between those that used no medication and those that used both antidepressants and sleep medication.

By comparing the body image scores from this study with other studies that used an adjusted BIS, it is clear that the average score from the present study was very high, namely 14.66. Studies among women who do not have FM but have other conditions, such as vaginal prolapse, genealogical problems, or cancer found BIS scores between 6.06 and 8.62 [16,20,21]. Similarly, average GHQ-12 scores in our sample (M = 7.08; SD = ±2.27) were higher than a national representative sample of the population in Belgium (M = 5.38; SD = ±3.45) [17], indicating increased mental health issues for FM women. This lower mental health has been shown to be associated with highly negative body images. The association between a disturbed body image and lower mental health has already been found in other populations [22], but rarely among individuals with FM (e.g., [10]).

In the present study, the role of fibromyalgia symptoms disappeared when taking into account mental health symptoms. As some fibromyalgia symptoms showed overlap with mental health problems, this may indicate that the association between FM and body image is mostly a result of mental health aspects of FM and not as a result of aspects such as physical pain or fatigue caused by FM [23]. The absence of a statistical relationship between FM symptoms and body image after controlling for mental health underlines this finding. However, this does not mean that other FM symptoms have no meaningful association with body image. A qualitative study showed that although FM is considered somatically not detectable, individuals with the disorder fear that their physical sensations such as joint stiffness, swelling, and other problems influence their appearance [4]. Considering that FM can indeed cause such somatic yet undetectable symptoms [1], it cannot be stated that it is purely an association between mental health and body image.

Another important insight from this study is that medication usage could be associated with body image, but only when not controlling for mental health. Thus, it seems that medication use does not have a clear association with body image.

There are a few strategies that clinicians/therapists can use to promote positive body image perception in FM patients. Firstly, different diets can help the body image of FM women [2]. Secondly, a study of people with rheumatoid arthritis demonstrated that education about body functionalities can result in improvements of body image and to decreased depressive symptoms [24].

This study is limited by the absence of indicators such as sexual dysfunction or BMI [5]. However, the aim of this study was to investigate the relationship between mental health symptoms, medication use, and body image. Including more predictors would have been difficult with the sample size used. However, if larger samples could be collected, it would be advised to also explore the roles of social context and identity. For example, as the study by Boyington et al. [4] shows, FM can affect social relationships and the identity of an individual. If these are affected, they will also affect mental health and perceived body image. The study is also limited by its cross-sectional design, which impedes the assessment of directions of relationships. For example, mental health problems may arise from FM, but it may also have been present before FM was identified, as mental health symptoms are not only a consequence of FM but are also predictors of FM [1]. Furthermore, despite having a fairly large sample for research on psychosocial consequences of FM, the problem remains that the representativeness of our sample for the wider population of people with FM, around 2–3% of the general population, is limited. Finally, the use of medications such as antidepressants was measured using a two-week period to allow easy estimations for participants, although the effectiveness of antidepressants is dependent on longer periods of use and on the dosage, both of which were not measured in our study.

This study has contributed to the psychosocial knowledge on fibromyalgia in several ways. First, it is one of the few that shows associations between mental health and body image in FM women, while controlling for medication use. Second, the study showed the importance of addressing body image among women with FM. Despite women with FM generally not suffering from clear signs of FM, their perception of their body image in the current study was very low. Third, the study showed that low body image seems to fit with our other knowledge on FM, namely that mental health issues that are generally higher among FM women are also associated with lower body images. Therefore, this shows the importance of viewing the health of people with chronic diseases as more than the symptoms of their diseases and that “health” is much broader than these symptoms [14]. To show the link between FM, mental health, and body image more, further research should implement a longitudinal design to investigate causal relations and add other variables in order to investigate the roles of social relations and sexuality.

## 5. Conclusions

Mental health issues and a lower body image are associated with each other, while medication use is not when controlling for other variables.

## Figures and Tables

**Table 1:** Characteristics of the sample.

Variables	N	%
Age		
18–27	2	1.9
28–37	16	15.5
38–47	31	30.1
48–57	39	37.9
58–67	13	12.6
68–77	2	1.9
When have you had a diagnosis of fibromyalgia?		
A month or less	6	5.8
Between 1 and 6 months ago	9	8.7
6 months and 1 year	8	7.8
1 and 5 years	29	28.2
More than 5 years	51	49.5
Variables	Mean	±SD
Medication use		
Antidepressants	1.81	0.924
Pain killer	2.41	0.76
Sleep medication	1.78	0.885
BIS	14.66	7.02
VASFIQ	49.17	10.48
GHQ-12	21.24	6.08

BIS = Body Image Scale; GHQ-12 = General Health Questionnaire-12; VASFIQ = Visual Analogue Scale Fibromyalgia Impact Questionnaire. Medication use: scale ranges from 1 to 3; BIS: scale ranges from 0 to 27; GHQ-12: scale ranges from 0 to 36; VASFIQ: scale ranges from 0 to 70.

**Table 2:** Associations of BIS with other variables.

	BIS	GHQ-12	VASFIQ	Anti-Depressants	Pain Medication	Sleep Medication	Age	Time Diagnosis
BIS	1	0.349 ***	0.296 **	0.133	0.077	0.272 **	−0.044	0.062
GHQ−12		1	0.529 ***	0.162	−0.049	0.037	−0.119	−0.154
VASFIQ			1	0.104	0.031	0.055	−0.058	−0.132
Antidepressant				1	0.171	0.303 **	0.006	−0.137
Pain medication					1	0.213 *	0.137	0.239 *
Sleep medication						1	−0.066	0.141
Age							1	0.344 **
Time diagnosis								1

BIS = Body Image Scale; GHQ-12 = General Health Questionnaire-12; VASFIQ = Visual Analogue Scale Fibromyalgia Impact Questionnaire. * *p* < 0.05, ** *p* < 0.01, *** *p* < 0.001.

**Table 3:** Comparison of different models in predicting body-image scale scores.

	B	±SE	R²	*p*
1			10	0.002
Constant	7.757	4.516		0.089
Time since diagnosis	1.091	0.551		0.051
VASFIQ	0.021	0.006		0.001 **
2			15.6	<0.001 ***
Constant	5.533	4.448		0.216
Time since diagnosis	1.243	0.537		0.023
VASFIQ	0.011	0.007		0.136
GHQ-12	0.306	0.111		0.007 **
3			14.3	<0.001 ***
Constant	6.006	4.467		0.182
Time since diagnosis	0.893	0.544		0.104
VASFIQ	0.02	0.006		0.002 **
Sleep medication	1.794	0.736		0.017 *
4			9.2	0.006 **
Constant	8	4.708		0.092
Time since diagnosis	1.117	0.57		0.053
VASFIQ	0.021	0.006		0.001 **
Pain medication	−0.174	0.897		0.846
5			10.7	0.003 **
Constant	6.11	4.678		0.195
Time since diagnosis	1.15	0.551		0.040 *
VASFIQ	0.02	0.006		0.002 **
Antidepressants	0.937	0.723		0.198
6			19.3	<0.001 ***
Constant	3.997	4.398		0.366
Time since diagnosis	1.05	0.531		0.051
VASFIQ	0.01	0.01		0.139
GHQ-12	0.292	0.109		0.009 **
Sleep medication	1.683	0.715		0.021 *
7			18.3	<0.001 ***
Constant	7.604	3.696		0.042 *
Time since diagnosis	0.997	0.534		0.065
GHQ-12	0.376	0.094		<0.001 ***
Sleep medication	1.704	0.719		0.020 *

BIS = Body Image Scale; GHQ-12 = General Health Questionnaire-12; VASFIQ = Visual Analogue Scale Fibromyalgia Impact Questionnaire. * *p* < 0.05, ** *p* < 0.01, *** *p* < 0.001.

**Table 4:** Comparison of participants who used no medication with participants who used antidepressants and sleep medication.

	No Medication (*n* = 35)	Antidepressants and Sleep Medication (*n* = 30)	*p*-Value
VASFIQ	30.43 (48.8)	36.00 (51.3)	0.236
BIS	27.67 (12.51)	39.22 (16.77)	0.014 *
GHQ-12	30.89 (20.0)	35.47 (21.67)	0.329

Mean ranks (averages in parentheses); Mann–Whitney test; * *p* < 0.05.

## Data Availability

Due to low cell risk (as our research population is small), using the entire database might result in the identification of participants.
